# Leveraging circulating microbial DNA for early cancer detection

**DOI:** 10.1016/j.trecan.2023.08.001

**Published:** 2023-11

**Authors:** Radhika Kataria, Saeed Shoaie, Anita Grigoriadis, Jonathan C.M. Wan

**Affiliations:** 1 Cancer Bioinformatics, School of Cancer & Pharmaceutical Sciences, Faculty of Life Sciences and Medicine, King's College London, London, UK; 2 Centre for Host-Microbiome Interactions, Faculty of Dentistry, Oral & Craniofacial Sciences, King’s College London, London, UK; 3 School of Cancer & Pharmaceutical Sciences, Faculty of Life Sciences and Medicine, King's College London, London, UK; 4 Science for Life Laboratory, KTH - Royal Institute of Technology, Stockholm, Sweden; 5 Breast Cancer Now Research Unit, School of Cancer and Pharmaceutical Sciences, Faculty of Life Sciences and Medicine, King’s College London, London, UK; 6 University College London Hospital, Euston Road, London, UK

**Keywords:** microbiome, circulating microbial DNA, liquid biopsy, cancer, early detection

## Abstract

Microbial cell-free DNA (microbial cfDNA) offers a minimally invasive approach for profiling the microbiome. Despite technical and biological challenges, the potential of microbial cfDNA for cancer detection is increasingly being evaluated using deep sequencing, novel laboratory approaches, and computational methods. Targeting the microbiome using liquid biopsies could improve early cancer detection.

## Exploring microbial cfDNA from liquid biopsies

Liquid biopsies offer a sensitive and minimally invasive approach for cancer detection and monitoring [1]. Tumour-derived markers such as mutations in **cell-free DNA (cfDNA)** (see Glossary) have conventionally been targeted, although in early-stage or residual disease settings, detection of low concentrations of **circulating tumour DNA (ctDNA)** can be challenging. In recent years, the exploration of non-human sources of cfDNA, originating from the **microbiome**, represents an emerging avenue for cancer detection. Whole-genome sequencing (WGS) provides a unique opportunity to investigate the microbiome using non-human sequencing reads obtained from large datasets [2].

Alterations in the human microbiome are known to occur in numerous diseases, which include cancer, inflammatory bowel disease, and autoimmune disorders [3]. In oncology, gut microbiome fluctuations have been found to influence the efficacy of immunotherapy [4]. Although our understanding of the role of the microbiome in carcinogenesis is incomplete, leveraging circulating microbial profiles in patients with disease is emerging as an exciting approach for minimally invasive cancer detection.

Metagenomic analyses are increasingly applied to characterise **microbial cfDNA** in the bloodstream using WGS [5,6]. This allows tumour-associated metagenomes made up of bacteria, viruses, and fungi to be deciphered [3]. Recently, minimally invasive profiling of microbial alterations using WGS coupled with a machine learning approach demonstrated the ability to identify individuals with cancer [5]. Alternative approaches for profiling microbial cfDNA or circulating RNA include targeted PCR detection of specific species or untargeted **16S rRNA** and shotgun sequencing. Last, non-nucleic acid-based approaches, such as the use of microbial proteins or epitopes in the circulation, might provide additional signal, which could be tested in parallel with nucleic acid-based approaches.

Leveraging microbial cfDNA for cancer detection presents both biological and technical challenges. Technically, detecting and quantifying low-abundance microbial cfDNA amidst the vast amount of human cfDNA (and contaminants) is difficult, even with current methods (Box 1). Furthermore, ongoing research highlights the complexity of the microbiome, which will be reflected in microbial cfDNA analysis.

Box 1 **Overcoming technical challenges of sequencing microbial cfDNA**

Characterising the microbial composition from WGS data is hindered by biological and technical noise, compounded by the majority of sequencing reads being of human origin, leaving few non-human sequences (Figure I). To increase the number of microbial reads sequenced, methods to enrich target DNA or deplete off-target DNA fragments should be considered. Using **single-stranded DNA (ssDNA) library preparation** allows microbial cfDNA fragments to be recovered with a greater efficiency and sensitivity, yielding a 71-fold increase in the relative genomic coverage of microbial species, in comparison to double-stranded DNA library preparation techniques [12]. Alternatively, given that microbial cfDNA is likely non-nucleosomal [1], if microbial cfDNA size profiles were established, size-based enrichment methods could increase yield.

External contaminants may be introduced into microbial cfDNA samples at a number of stages: during sample collection, from laboratory surfaces, and/or from reagents. Internal cross-contamination may occur by sample mixing, such as during DNA extraction or sequencing [13]. To account for external contamination sources, ‘negative-blank’ controls of reagents can be run alongside the biological samples; to identify internal contaminants, synthetic DNA sequences can be spiked into each sample [14].

Often *in silico* decontamination methods ‘blacklist’ contaminating microbes form downstream analyses. For example, the assessment of batch effects, such as by sequencing centre or by reagent kit, may identify confounding taxa or could even identify signatures of contamination. Alternatively, bacterial genus read count can be compared against sample DNA concentrations in a statistical framework to identify contaminating microbes [13]. Hard filters can be applied to remove published contaminants, although contaminating species may often overlap with cancer-associated microbes. Recent approaches, based on source tracking, seek to model the contribution of each contaminating source to the overall microbial profile, resulting in improved classification of melanoma versus control using microbial cfDNA [15].

Computational methods for identifying microbial taxa present in sequencing data are rapidly improving, although challenges remain. Certain bioinformatics tools rely on **k-mer based classification** models, whose accuracy may be hampered by the short fragment size of cfDNA. Short reads producing short k-mers could result in false-positive microbial characterisation or limit the resolution of taxonomic classification, particularly for highly repetitive k-mers. With greater sequencing depth, *de novo* assembly of shorter fragments might be used to improve the specificity of microbial classifications. Other approaches include alignment-based methods, such as SHOGUN, used by Poore *et al*. [5], which uses a statistical method for estimating taxon abundance.

**Figure I:**
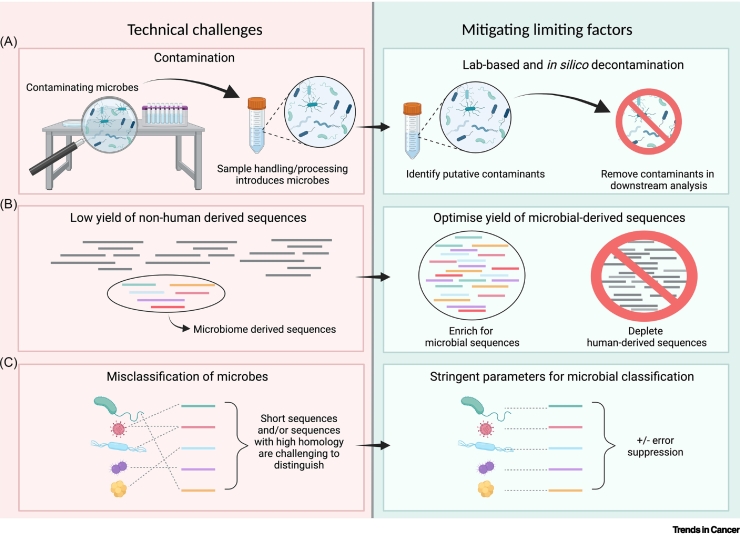
Technical challenges associated with analysing microbial cfDNA from liquid biopsies and approaches to mitigate these limitations. (A) Contaminating microbes from the environment can be introduced into liquid biopsy samples during the sample preparation, handling, and processing steps. These may be addressed using negative blank controls sequenced alongside the biological sample to remove contaminants, and bioinformatic filters may be applied. (B) Low yield of microbial cfDNA sequences due to the majority of reads being of human origin. The yield of microbially derived sequences can be optimised by using ultrashort fragment enrichment techniques, or alternatively human-derived sequences might be depleted. (C) Finally, short sequences or sequences with high homology may lead to the misclassification of microbes. Classification also depends on the breadth of the microbial reference database used. Using stringent parameters for microbial classifications with a margin for error suppression can increase the accuracy for microbial characterisation.

Alt-text: Box 1

This article aims to explore the potential for early cancer detection using microbial cfDNA. We discuss the application of laboratory and computational metagenomic techniques for profiling microbial cfDNA, including the role of data science and machine learning. Furthermore, we address the limitations associated with these approaches and discuss the future prospects of this rapidly evolving field.

## Leveraging microbial cfDNA for early cancer detection

Different tumour types have been shown to have different intratumour microbial profiles [7]. Correspondingly, patients’ microbial cfDNA profiles have been found to differ by cancer type [5]. Microbial cfDNA profiles enabled cancer detection in individuals with a variety of cancer types even when genomic alterations were not as prevalent, such as in stages I and II tumours [5], and between premalignant and malignant disease in oesophageal cancer [8]. This suggests that microbial cfDNA alterations may occur early in cancer development. In the future, the sensitivity of microbial cfDNA for early cancer detection should be compared against ctDNA and may boost detection rates when used together (Box 2).

Box 2 **Can cancer detection be enhanced by targeting both microbial cfDNA and ctDNA?**

Detection of early-stage cancer using liquid biopsy has traditionally been challenging due to low concentrations of ctDNA [1]. To mitigate this sampling challenge for rare mutant molecules, multiple tumour mutations or epigenetic loci should be targeted in parallel [1]. Alternatively, multiomic approaches that combine multiple markers may be used.

Existing ctDNA assays detect tumour-derived genomic alterations; yet, often they do not take into consideration microbial cfDNA in parallel. Microbial cfDNA is released by multiple sites within the body that compose the microbiome, some of which may be from intratumour bacteria and fungi [7]. Despite the abundance of microbial cells within the body, microbial cfDNA represents only a small percentage of WGS data obtained from plasma.

The microbiome contains thousands of taxa, many of which can be altered in cancer. Analogous to a large number of tumour reporters monitored by existing ctDNA assays, each taxon may serve as a data point that might be used for classification using machine learning. Thus, small changes in any individual taxon, when aggregated across the whole microbial cfDNA profile, may enable cancer detection, even though the absolute abundance of microbial cfDNA may be low.

Given that microbial cfDNA data may be obtained alongside human WGS data, it may be possible in the future to generate machine learning models that incorporate both human and non-human markers of disease. Microbial cfDNA should be interrogated in its broadest sense for maximal sensitivity, incorporating analysis of bacteria, fungi, and viruses. This might boost sensitivity for early cancer detection, in keeping with the trend toward multiomic liquid biopsy approaches. Retrospective analysis of existing plasma WGS datasets may be challenging, however, due to the requirement for microbial cfDNA control samples and characterisation of local contaminants.

Alt-text: Box 2

Detection of microbial cfDNA from pathogenic microbes implicated in oncogenesis may provide an opportunity for targeted sequencing panels for cancer detection. Previously, plasma screening for the Epstein-Barr virus (EBV) identified nasopharyngeal carcinomas in asymptomatic individuals in Hong Kong with high sensitivity and specificity [9]. To reduce false-positive results, only patients with serially positive EBV results were taken forward. In the future, similarly, targeted deep sequencing of *Helicobacter pylori*, *Escherichia coli*, or *Fusobacterium nucleatum* amplicons might indicate increased cancer risk, although the positive predictive value would need to be optimised through strategies such as testing for persistently raised species or inclusion of other relevant species. Such targeted screening approaches may be clinically valuable in high-risk populations, although whether cancer-associated microbial targeted panels could be sufficiently sensitive for pan-cancer detection remains to be seen.

## Biological challenges

The composition of an individual’s microbiome is influenced by factors including age and gender [5] as well as lifestyle and diet. These biological factors could in turn affect microbial signatures reflecting disease states and may be considered for normalisation in analyses of microbial cfDNA. For example, Poore *et al*. [5] normalised for age and gender. Given the complexity of the microbiome, there are likely further unknown confounders, and, as such, patient and control groups require careful matching as well as large sample sizes to mitigate interindividual variation.

Microbial cfDNA profiles may reflect dynamic changes due to subclinical or clinical infections, as demonstrated in a cohort of patients following solid-organ transplant [10]. The magnitude of such changes would likely be smaller in nonimmunosuppressed patients, although the presence of any intercurrent infection, particularly those that affect the organ most proximal to the tumour, should be considered. Separately, the effect of treatments on microbial cfDNA, particularly antibiotics, should be explored. Serial liquid biopsy samples from the same individual might enable the identification of transient changes [10].

Detection rates of cancers using ctDNA vary by cancer type [1], likely related to biological differences in rates of ctDNA release from each cancer. Similarly, we speculate that detection rates based on microbial cfDNA would likely also differ by cancer type: they may differ in concentrations of intratumour microbes, rate of release of microbial DNA from their intratumour microbiome, and/or in enzymatic processing of released DNA. Given the large number of bacteria within the gut, tumours whose aetiology is associated with alterations to their gut microbiome might be more amenable to microbial cfDNA-based early cancer detection, though further study is required.

## Concluding remarks and future directions

Initial studies in the field of microbial cfDNA show promise for cancer detection [5,8,9]. Next, the application of data science and interpretable machine learning models might allow the identification of specific microbial communities or signatures associated with certain cancer types, and those driving classification. In contrast to tissue biopsies, which may only sample intratumour microbes, liquid biopsies may capture a broader representation of the microbiome, including nontumour microbes, which could theoretically be altered by disease.

The source of microbial cfDNA requires further characterisation: although tumours contain intracellular bacteria [7], the relative contribution of intratumour bacteria versus microbes at nontumour sites is not clear. Analyses of circulating bacterial and fungal DNA in individuals with early-stage cancers found that classification based on tumour-derived species alone performed similarly to a 26-fold larger database of species [2]. Although this suggests a significant contribution of cfDNA from species present within tumours, these species may also be present outside of tumours. This is evidenced by Narunsky-Haziza *et al.* [2], whereby unsupervised clustering revealed the same microbial species within tumours that were also found in normal adjacent tissue. Lastly, associations between gut microbiome composition and immunotherapy response have been reported in patients with kidney and lung cancers, indicating that an imbalance in gut flora may correlate with impaired immune cell activity [4]. Depending on the contribution of the gut microbiome to microbial cfDNA, profiling both the gut microbiome and plasma may provide information on the efficacy of immunotherapies.

Interestingly, microbial cfDNA sequences have been identified with no homology to existing bacterial species, suggesting the presence of novel microbial species that may be accessible by liquid biopsy sampling only [11], although it is unclear where such microbes reside. We suggest that comparing microbial cfDNA profiles both before and after surgery may allow contributions of intratumour bacteria to be deciphered relative to the wider microbiome. However, the confounding effects of surgery itself and antimicrobial therapies need to be considered.

Microbial cfDNA may also be used for the early detection of other noncancer diseases. Sequencing a large number of healthy samples with careful **decontamination** may enable the characterisation of a baseline microbial cfDNA profile, potentially enabling disease detection by identifying small perturbations in microbial cfDNA abundance. With sufficient data, future work exploiting microbial cfDNA profiles using machine learning might enable risk profiling or patient stratification to personalised therapies. Longitudinal liquid biopsy studies of microbial cfDNA in healthy individuals might be particularly suited to characterising the background variance of microbial cfDNA profiles and their causes.

In summary, microbial cfDNA provides an exciting opportunity to study and detect cancer by leveraging the microbiome. Whilst the analysis of microbial cfDNA requires careful consideration to overcome biological and technical challenges, it has the potential to offer valuable information for early cancer detection. Therefore, multidisciplinary collaboration over the coming years will be paramount to realising the potential of this emerging cancer diagnostic.

## Glossary

**16S rRNA sequencing**: a method to analyse the composition and diversity of microbial communities by sequencing rRNA.
**Cell-free DNA (cfDNA)**: DNA fragments released by cells extracellularly, which may be identified in the bloodstream.
**Circulating tumour DNA (ctDNA)**: fragments of DNA released by tumour cells into the bloodstream, which can contain mutations.
**Decontamination**: process of removing or reducing the presence of erroneous microbial signals in data, which may be performed *in vitro* or *in silico*.
**k-mer classification**: the process of classifying sequences based on the presence or absence of specific subsequences of length k (k-mers) within the sequence to be classified.
**Microbial cell-free DNA (microbial cfDNA)**: DNA fragments released by microbes in the human body, which may be identified in the bloodstream.
**Microbiome**: the aggregate of microorganisms in the body, such as bacteria, fungi, and viruses.
**Single-stranded DNA (ssDNA) library preparation**: a library preparation method which improves recovery of <100 bp cfDNA fragments by leveraging single-stranded ligation chemistry.
